# Phospholipid codes at virus-host membrane interfaces: mechanisms and therapeutic opportunities in animal viral diseases

**DOI:** 10.3389/fcimb.2026.1953135

**Published:** 2026-09-29

**Authors:** Lei Tan, Guoyuan Wen

**Affiliations:** 1 Shanghai Veterinary Research Institute, Chinese Academy of Agricultural Sciences, Shanghai, China; 2 Institute of Animal Science and Veterinary Medicine, Hubei Academy of Agricultural Sciences, Wuhan, Hubei, China

**Keywords:** animal viral diseases, host-targeted antivirals, lipid rafts, membrane-state transitions, phosphatidylserine, phospholipid code, replication organelles, virus-host membrane interface

## Abstract

Animal viruses depend on host membranes at every stage of infection, yet membrane lipid states are often treated as background rather than as causal determinants of virus-host interactions. This Review develops a phospholipid-code framework in which headgroup identity, acyl-chain chemistry, leaflet topology, and lipid flux define infection-stage-specific membrane states. Evidence from Newcastle disease virus, infectious bronchitis virus, avian influenza virus, porcine reproductive and respiratory syndrome virus, African swine fever virus, Senecavirus A, Japanese encephalitis virus, and porcine epidemic diarrhea virus shows that these states can control attachment, endosomal progression, growth of replication organelles, viral protein allostery, budding, virion infectivity, ferroptotic tissue injury, and delivery of vaccines or antivirals. Comparative evidence from human and plant virus systems is used only to identify transferable membrane mechanisms that require validation in animal-virus models and natural host species. The framework distinguishes viral lipid demand from host defense and tissue damage and explains why total-cell lipidomics or broad metabolic inhibitors rarely establish causality. We argue that the most promising interventions will target viral protein-lipid interfaces, virus-specific recruitment of lipid-transfer machinery, transient leaflet transitions, virion envelopes, or pathological lipid oxidation rather than globally suppressing essential lipid metabolism. Species-relevant primary cells, organoids, virion lipidomics, topology-resolved assays, acute perturbation, rescue experiments, and animal challenge models will be required to translate causal phospholipid biology into safe and practical antiviral strategies for livestock and poultry health.

## 1 Introduction

Viruses are commonly described through protein-centered features: receptor-binding proteins, fusion proteins, polymerases, capsid proteins, glycoproteins, and immune antagonists. That description is necessary but incomplete. Every stage of infection also takes place in a chemically and topologically defined membrane environment. A virion must attach to a permissive surface, cross a membrane whose composition supports fusion or penetration, construct intracellular membrane niches for genome replication, assemble at selected membrane domains, and leave the producer cell with an envelope or membrane-associated cargo that influences the next round of infection. Here, the virus-host membrane interface refers to the plasma-membrane, endosomal, organellar, and virion-membrane contacts at which viral proteins, host receptors or machineries, and local lipids interact during attachment, entry, replication, assembly, budding, immune sensing, or tissue injury.

This issue is particularly relevant to animal viral diseases. Newcastle disease virus (NDV), infectious bronchitis virus (IBV), avian influenza virus, porcine reproductive and respiratory syndrome virus (PRRSV), African swine fever virus (ASFV), Senecavirus A (SVA), Japanese encephalitis virus (JEV), and porcine epidemic diarrhea virus (PEDV) infect different species and tissues, yet all must negotiate host membrane identity. Their dependence on host lipid organization creates mechanistic links between viral entry, replication, assembly, inflammatory injury, and therapeutic vulnerability. It also creates practical constraints: a useful veterinary intervention must preserve essential membrane functions in the target animal, work in the relevant primary cell or tissue, and remain compatible with production-scale delivery, food-animal safety, and field use.

We use the term phospholipid code to describe an infection-stage-specific membrane state defined by four coupled variables: headgroup identity, acyl-chain chemistry, leaflet topology, and lipid flux. A code is more precise than a change in bulk lipid abundance because it specifies which lipid species is present, in which compartment and leaflet, at which stage of infection, and which protein or pathway responds to it. To distinguish functionally different responses, we classify code readers into three groups. Effector readers directly execute a lipid-dependent event, such as a fusion protein or the SVA 3C protease. Recruitment readers organize or supply a membrane platform, including viral nonstructural proteins and host lipid-transfer systems that build replication organelles. Sensor readers detect an exposed or chemically modified lipid state and initiate uptake or immunity, exemplified by TIM/TAM-family receptors and innate signaling pathways. The same lipid state can therefore produce different outcomes according to reader class, compartment, cell type, and timing. This framework also helps distinguish three interpretations of infection-associated lipid remodeling: viral demand, host defense, and tissue damage.

The review is centered on animal viruses and translation to natural host species. Evidence from human or plant virus systems is retained only when it establishes a transferable membrane mechanism that has not yet been resolved with comparable mechanistic depth in an animal-virus system. Such comparative evidence is explicitly identified and is not treated as a substitute for validation in avian, porcine, bovine, or other relevant host systems. The main argument is that therapeutically useful targets are more likely to be infection-specific membrane-state transitions or virus-specific recruitment interfaces than global lipid metabolism.

The phospholipid code operates within a multimolecular interface rather than independently of proteins or glycans. In ASFV infection, membrane trafficking is coupled to cytoskeletal transport: viral p54 binds the dynein motor complex (Alonso et al., 2001), and movement of newly assembled virions from perinuclear factories toward the plasma membrane depends on microtubules and conventional kinesin (Jouvenet et al., 2004). In PRRSV, cell-surface heparan sulfate supports early attachment and enhances subsequent engagement of sialoadhesin, demonstrating sequential cooperation between a glycan and a protein receptor (Delputte et al., 2005). These examples illustrate that local lipid states should be interpreted as permissive or instructive layers that cooperate with receptors, cytoskeletal motors, membrane-trafficking machinery, and glycans rather than as autonomous determinants of infection.

## 2 Phospholipid variables that define virus-host membrane interfaces

### 2.1 Headgroup identity and compartmental context

Phosphatidylcholine (PC), phosphatidylethanolamine (PE), phosphatidylserine (PS), phosphoinositides, cardiolipin, and bis(monoacylglycero)phosphate (BMP; also termed lysobisphosphatidic acid, LBPA) are not interchangeable building blocks. PC contributes much of the bilayer mass, whereas PE favors negative curvature and can become a receptor-visible signal when externalized. PS combines anionic charge with immunological meaning because externalized PS can engage TIM and TAM family receptors, promote efferocytosis-like uptake, influence coagulation, or signal cellular damage. Phosphoinositides are low-abundance but high-information lipids that recruit proteins and define organelle identity. BMP/LBPA is enriched in late endosomes and supports membrane deformation and late fusion steps.

The importance of headgroup identity is illustrated by influenza virus membrane fusion. Productive infection is reduced when influenza A virus is forced to fuse at the plasma membrane rather than through its physiological endosomal route (Mannsverk et al., 2025). In single-virus assays, BMP/LBPA increases the probability that hemifusion progresses to fusion-pore formation (Mannsverk et al., 2022). These findings demonstrate that low pH is not sufficient: the endosome provides a lipid-defined reaction environment. Comparative coronavirus studies similarly show that anionic phospholipids can stabilize fusion-peptide intermediates (Villalaín, 2023; Singh et al., 2024). The mechanistic lesson for animal viruses is that receptor binding and proteolytic activation should be interpreted together with the native lipid state of the target membrane.

### 2.2 Acyl-chain chemistry, leaflet topology, and lipid flux

Acyl-chain length and unsaturation regulate membrane thickness, order, packing defects, curvature stress, and susceptibility to oxidation. These properties affect fusion protein insertion, budding, membrane protein conformation, and lipid peroxidation. In JEV infection, ferroptotic phospholipid peroxidation contributes to neuronal damage and neuroinflammation (Zhu et al., 2024). In influenza A virus-induced acute lung injury, indoleamine 2, 3-dioxygenase 1 drives epithelial ferroptosis (Zheng et al., 2025). These examples show that acyl-chain chemistry can become a determinant of pathology rather than a passive lipidomic signature.

Leaflet topology adds a second layer of information. PS and PE can be abundant in a membrane without being exposed to extracellular receptors. Their externalization changes the biological meaning of the membrane. NDV provides unusually direct evidence: the virus exploits the ATP11c–CDC50A phospholipid flippase complex during infection and acquires PS during budding, generating progeny particles with enhanced infectivity (Hou et al., 2025; Zhang et al., 2025). The effect is therefore not explained by total PS abundance; it depends on where PS is positioned and whether the next target cell can read it.

The fourth variable is lipid flux. Replication organelles and viral factories require continuous delivery, redistribution, and remodeling of membrane material. Positive-strand RNA viruses provide the clearest structural examples, but large DNA viruses such as ASFV also reorganize cholesterol and membrane traffic. Bulk lipid-transfer proteins, membrane contact sites, autophagy factors, vesicle trafficking, phospholipases, and lipid-droplet turnover can all become supply routes. Evidence from tombusvirus shows that ATG2 can be recruited to deliver bulk phospholipid into replication organelles (Kang et al., 2024), whereas formation of coronavirus replication organelles requires coordinated viral membrane proteins and phosphoinositide-linked lipid exchange (Ma-Lauer et al., 2024; Li et al., 2025). These comparative systems define testable mechanisms for animal viruses of veterinary importance even when the exact host factor remains unknown (**Figure 1**).

**Figure 1:**
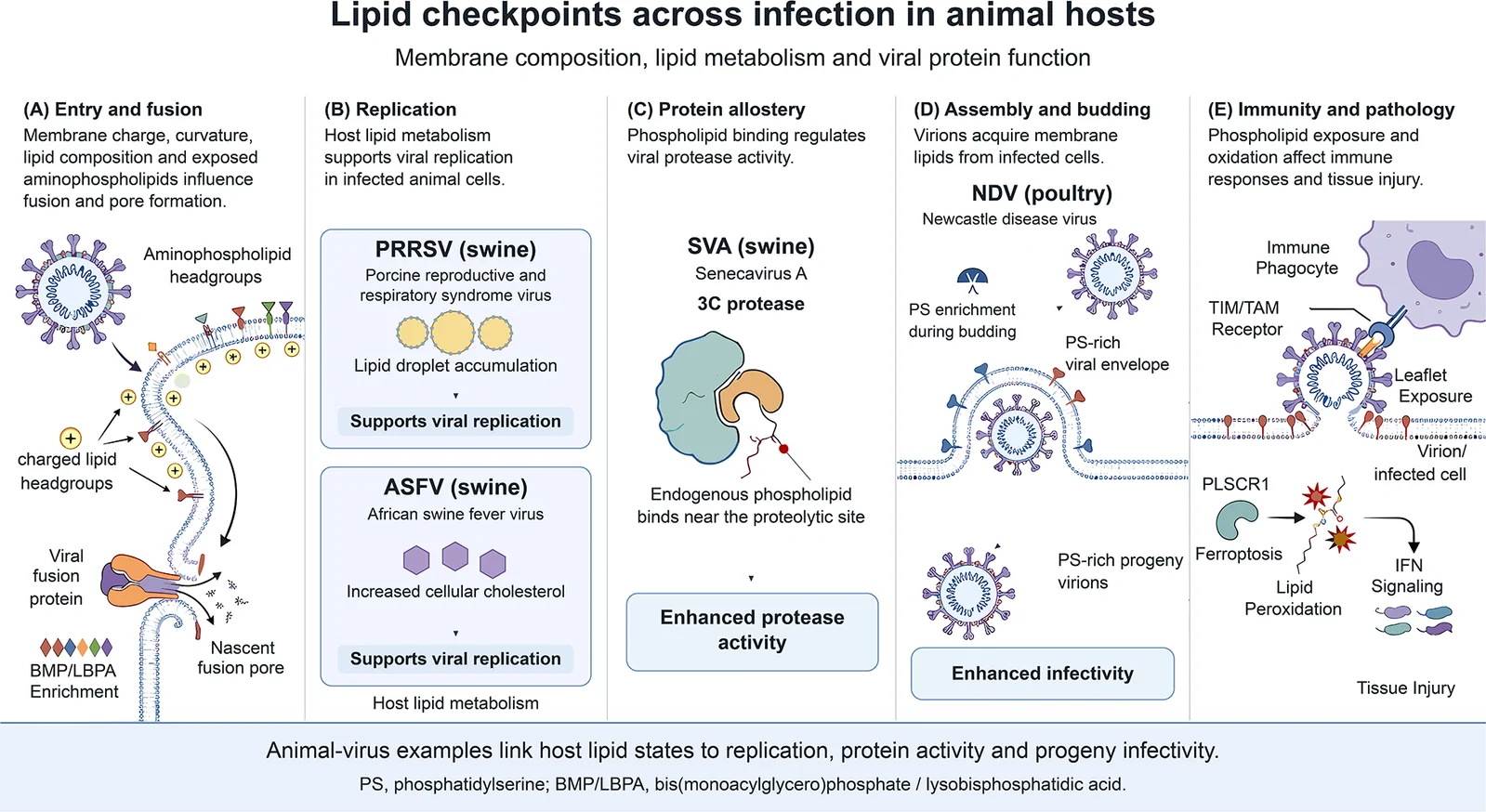
Membrane-state checkpoints across the animal-virus life cycle. **(A)** Membrane charge, curvature, lipid composition and aminophospholipid exposure influence viral entry and fusion. **(B)** Examples from swine viruses illustrate how host lipid metabolism supports replication: porcine reproductive and respiratory syndrome virus (PRRSV) infection promotes lipid droplet accumulation, while increased cellular cholesterol supports African swine fever virus (ASFV) replication. These examples do not establish direct phospholipid transfer from lipid droplets to replication organelles; cholesterol is included as a distinct membrane lipid relevant to the broader metabolic context. **(C)** An endogenous phospholipid binds near the proteolytic site of Senecavirus A (SVA) 3C protease and enhances its activity. **(D)** Newcastle disease virus (NDV) acquires phosphatidylserine (PS) during budding, generating PS-rich progeny virions with enhanced infectivity. **(E)** Phospholipid exposure and oxidation contribute to immune recognition, signaling and tissue injury. Panels **(A, E)** summarize general processes; the explicitly named viral examples in panels **(B–D)** are pathogens of swine or poultry. Drawings are schematic and are not to scale. BMP/LBPA, bis(monoacylglycero)phosphate/lysobisphosphatidic acid; TIM, T-cell immunoglobulin and mucin domain; TAM, TYRO3, AXL and MERTK receptor family; PLSCR1, phospholipid scramblase 1; IFN, interferon.

## 3 Entry and fusion: animal viruses select membrane states, not arbitrary surfaces

### 3.1 Avian respiratory viruses

Influenza A virus provides a mechanistic example in which endosomal lipid identity influences productive entry. The fusion protein supplies conformational energy, but the target bilayer must accommodate curvature, local thinning, hydration defects, and pore expansion. BMP/LBPA-dependent fusion-pore formation provides a mechanistic explanation for why the physiological endosomal route outperforms forced plasma-membrane fusion (Mannsverk et al., 2022; 2025). This distinction matters for poultry infection because epithelial differentiation, endosomal maturation, and surfactant composition differ across the upper and lower respiratory tract. Whether these membrane requirements are conserved in avian influenza virus should therefore be tested directly in differentiated avian respiratory cells or organoids with native endolysosomal lipid organization, rather than inferred from transformed mammalian cell lines.

IBV, an avian coronavirus, illustrates a different entry checkpoint. Cholesterol depletion or disruption of lipid rafts before infection reduces IBV attachment, whereas later genome replication and virion release are less sensitive in the same experimental system (Guo et al., 2017). Thus, a lipid domain can function as an attachment platform without being a universal requirement for every subsequent stage. This stage specificity is critical for drug interpretation: a compound that disrupts rafts may appear broadly antiviral in a pre-treatment assay but may have little effect once the virus has entered. Time-of-addition experiments and rescue with cholesterol or defined lipids are therefore essential.

Accordingly, pretreatment with a raft-disrupting compound should not be interpreted as evidence that the entire IBV life cycle is lipid-raft dependent. Time-of-addition studies and cholesterol-repletion experiments are needed to determine whether an observed antiviral effect is confined to attachment/entry or extends to post-entry stages.

### 3.2 PRRSV and the dual role of cellular and viral membrane domains

PRRSV uses lipid rafts on both sides of the virus-host interface. Cellular raft disruption inhibits entry, and the receptor CD163 and viral glycoproteins are associated with raft fractions. Raft integrity also supports viral RNA production, while cholesterol depletion from the viral envelope damages particle integrity and reduces infectivity (Yang et al., 2015). The same membrane feature therefore contributes to receptor-proximal entry, replication-associated organization, and virion stability. This multi-stage dependence makes PRRSV a strong model for studying how a single lipid perturbation can produce different phenotypes depending on timing and compartment.

Phospholipase C signaling provides an additional cellular mechanism. Pharmacological inhibition of phospholipase C reduces PRRSV infection in cultured cells and alters infection-associated signaling, including p38 MAPK activation (Yuan et al., 2019). Because phospholipase C simultaneously changes signaling lipids, calcium responses, and membrane trafficking, future studies should separate catalytic lipid turnover from downstream inflammatory effects. Genetic perturbation of specific phospholipase isoforms, acute lipid sensors, and lipid rescue experiments would help identify which branch of the pathway is required for viral entry or replication.

### 3.3 ASFV entry through cholesterol-dependent endosomal progression

ASFV enters macrophages through endocytic pathways and undergoes stepwise disassembly in maturing endosomes. Cholesterol flux is required for virions to progress through late endosomes and reach the cytosol. Experimental blockade of endosomal cholesterol efflux causes cholesterol accumulation, impairs fusion or membrane penetration, and retains virions in endosomal compartments (Cuesta-Geijo et al., 2016). ASFV also increases cholesterol uptake and redistributes free cholesterol toward viral replication sites. The entry requirement is therefore linked to a broader reorganization of host lipid traffic.

This mechanism provides a useful caution for host-directed therapy. Complete inhibition of cholesterol synthesis or trafficking would be poorly tolerated and could alter macrophage function. A more selective strategy would interfere with the virus-specific recruitment, timing, or subcellular routing of cholesterol while preserving basal homeostasis. Compounds that accumulate in late endosomes may also exert pleiotropic effects on acidification, proteolysis, and membrane traffic; antiviral phenotypes must therefore be linked to a defined membrane transition rather than attributed to “cholesterol depletion” alone.

ASFV and IBV illustrate why a shared label such as cholesterol dependence can conceal distinct membrane-state requirements. For ASFV, dynamic cholesterol flux through maturing endosomes is required for cytosolic penetration, and cholesterol uptake or redistribution is also increased at later replication sites; the relevant state is therefore a flux-dependent endosomal and replication program in macrophages. By contrast, in the IBV Beaudette cell-culture system, cholesterol-rich rafts act mainly during attachment/entry, whereas later replication and release are less sensitive. Thus, the IBV requirement is more strongly stage-restricted and surface-domain focused (Cuesta-Geijo et al., 2016; Guo et al., 2017). These differences are likely to interact with target-cell identity—porcine macrophages for ASFV and avian respiratory epithelium for IBV—and receptor organization. They provide a testable route by which membrane states could contribute to tissue tropism or host range, but this hypothesis requires validation in natural host tissues.

## 4 Replication organelles and viral factories as lipid-consuming reaction chambers

Positive-strand RNA viruses remodel host membranes into replication organelles that concentrate viral RNA, polymerases, host factors, and metabolites while limiting exposure to innate sensors (Stancheva and Sanyal, 2024). These structures require both membrane area and membrane identity. Viral proteins bend, pair, invaginate, or tether membranes, but membrane-shaping activity is not equivalent to membrane supply. The ATG2–tombusvirus system demonstrates that a bridge-like lipid-transfer protein can be directly recruited to feed a growing replication organelle (Kang et al., 2024). HCV NS5A provides a complementary principle: phospholipid-rich environments can stabilize specific viral protein conformations and link protein folding to membrane remodeling (Bulankina et al., 2026).

For animal viruses, the most important implication is methodological. A reduction in replication after inhibiting a lipid-transfer protein can arise from insufficient membrane mass, loss of a specific headgroup, altered curvature, defective organelle contacts, or generalized organelle stress. These possibilities require different therapeutic strategies. Acute degron systems, pulse–chase lipid labeling, organelle-resolved lipidomics, and structure-guided mutants can distinguish a bulk supply function from a lipid-reader interaction.

### 4.1 PRRSV-induced cellular lipid droplets and metabolic rerouting

PRRSV increases lipid-droplet accumulation and redirects lipid metabolic pathways in ways that support viral replication (Yang et al., 2023). Lipid droplets should not be treated simply as neutral-lipid storage organelles. Their phospholipid monolayer recruits enzymes and trafficking proteins, and their physical contacts with the endoplasmic reticulum, mitochondria, and other organelles can redistribute fatty acids and membrane precursors. The key unresolved question is whether PRRSV requires lipid droplets as material reservoirs, signaling platforms, sites of protein localization, or regulators of lipotoxic stress. Distinguishing these functions will require perturbations that alter droplet biogenesis, lipolysis, or surface proteins without causing global energy failure.

### 4.2 Phospholipid regulation of the SVA 3C protease

SVA extends the lipid-reader concept from membrane assembly to viral enzymology. Its 3C protease binds an endogenous phospholipid near the proteolytic site, and the interaction enhances catalytic activity (Zhao et al., 2023). This is a direct example of phospholipid-dependent viral protein allostery in an economically relevant swine virus. It suggests that lipid-binding pockets or lipid-stabilized conformations could be targeted without globally suppressing the host lipid class. Structural validation, viral mutants that selectively disrupt lipid binding, and *in vivo* fitness studies are necessary to determine whether the interface offers a practical therapeutic window.

SVA proteins also induce ferroptosis in ways that can support replication (Song et al., 2025). Together, these findings connect phospholipid recognition, viral enzymatic activity, and oxidized phospholipid chemistry. They also show why a lipid pathway can be proviral at one stage and pathogenic at another. Therapeutic design must specify whether the goal is to inhibit viral replication, protect tissue, or both.

### 4.3 ASFV replication factories and host cholesterol metabolism

Recent transcriptomic and biochemical evidence indicates that ASFV infection disrupts host lipid metabolism and that increased intracellular cholesterol supports viral replication, whereas inhibition of cholesterol biosynthesis reduces viral yield (Chu et al., 2025). This complements the earlier entry data and suggests that ASFV has temporally distinct cholesterol requirements: controlled endosomal flux during entry and increased supply or redistribution during the formation of perinuclear cytoplasmic replication factories, where ASFV genome replication and particle assembly are concentrated. A single endpoint measurement cannot resolve these stages. Time-resolved perturbation is needed to distinguish entry blockade from late metabolic restriction.

## 5 Leaflet asymmetry, apoptotic mimicry, and the virion as an inherited lipid phenotype

Enveloped viruses do not sample host membranes randomly. They bud from selected domains, reorganize proteins and lipids, and inherit a surface chemistry that can influence stability, receptor use, immune sensing, and tissue pathology. The viral envelope is therefore a non-genetic phenotype shaped by the producer cell. NDV provides a direct example among the animal viruses considered here. The virus uses calcium signaling and phospholipid-handling machinery to expose and acquire PS during budding, and PS-rich progeny particles exploit PS-recognizing receptors to enhance infection (Hou et al., 2025). Budding is thus preparation for the next entry event.

This mechanism also reframes host-range and model-selection questions. Human TIM1 binds PE as well as PS more effectively than rhesus or mouse orthologs, producing species-dependent differences in viral uptake and efferocytosis (Zhang et al., 2024). Although this comparison does not directly model infection in a livestock species, it demonstrates that a lipid-mediated entry mechanism differs across receptor orthologs. Animal-virus studies should therefore test the relevant avian, porcine, bovine, or other species receptor rather than extrapolating from human or murine proteins.

PS exposure is not always proviral. PS-exposing extracellular vesicles in body fluids can compete for PS receptors and act as decoys against apoptotic-mimicry viruses (Groß et al., 2024). Likewise, scramblase-family proteins can have opposite effects depending on membrane location and signaling context. ANO6-mediated PS scrambling can facilitate coronavirus spike-induced fusion (Sim et al., 2022), whereas PLSCR1 supports interferon-linked restriction mechanisms, including IFN-λ receptor signaling during influenza A virus infection (Yang et al., 2025). The relevant unit is therefore not “PS exposure” or “scramblase activity” in isolation, but a defined topology, time point, receptor, and cell type (**Figure 2**).

**Figure 2:**
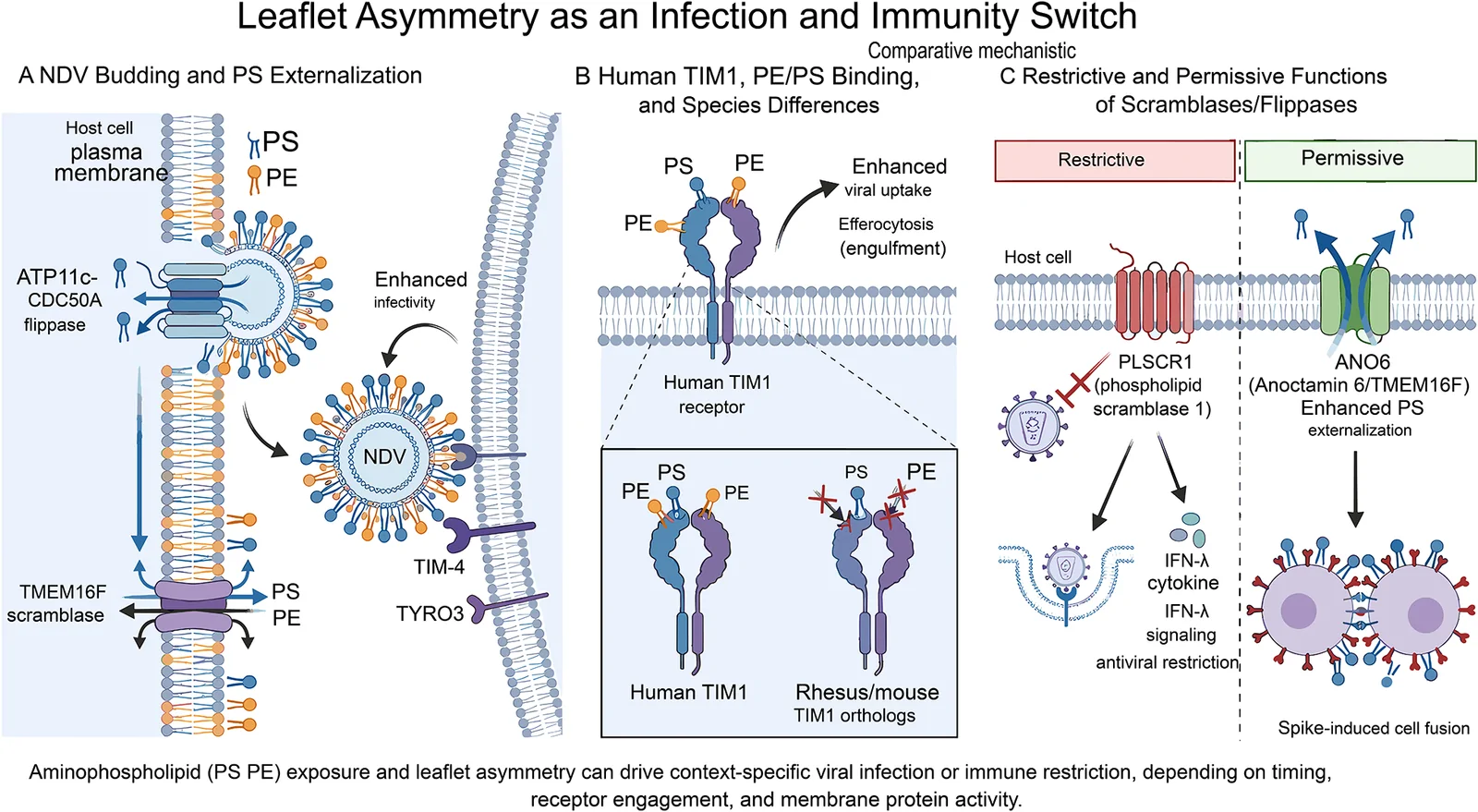
Leaflet asymmetry as an infection and immunity switch. Panel **(A)** summarizes direct NDV evidence for PS acquisition during budding and enhanced progeny infectivity. Panels **(B, C)** are explicitly marked as comparative evidence: TIM1 ortholog studies illustrate species-dependent PE/PS recognition, while PLSCR1 and ANO6 studies show that scrambling pathways can be restrictive or permissive depending on membrane location, timing, receptor context, and downstream signaling. These comparative systems are used to generate hypotheses for animal-virus studies rather than as direct livestock validation.

## 6 Infection-induced lipid remodeling: viral demand, host defense, or tissue damage?

Lipidomics has expanded rapidly in virus-host research, but abundance changes are mechanistically ambiguous. An increase in a phospholipid species may represent material consumed by a replication organelle, a host attempt to repair membranes, a systemic inflammatory response, or the release of lipids from dying tissue. Causal interpretation requires localization, timing, perturbation, and rescue (**Figure 3**). A plasma signature may be useful as a biomarker without being a suitable therapeutic target.

**Figure 3:**
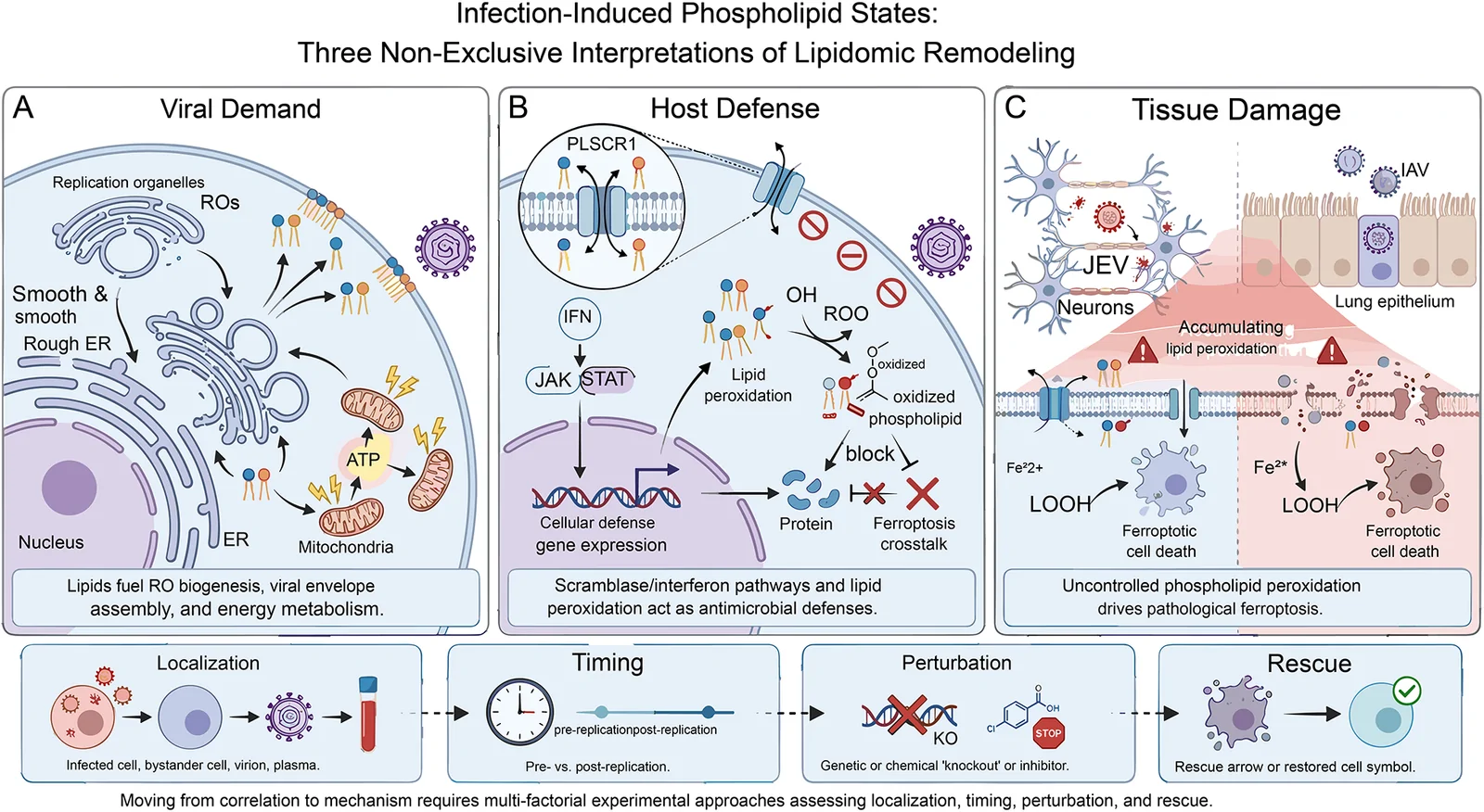
Infection-induced phospholipid states have three non-exclusive interpretations. Lipidomic remodeling may reflect viral demand, host defense, or tissue damage. Panels **(A, B)** schematically summarize general mechanisms of lipid utilization and membrane-linked host defense and are not intended to assign these pathways to a specific virus. Panel **(C)** highlights animal/zoonotic pathology examples, including JEV-induced neuronal injury and influenza A virus (IAV) induced lung injury in experimental models, in which phospholipid peroxidation contributes to ferroptotic tissue damage. Causal interpretation requires localization, temporal ordering, acute perturbation, and mechanistically specific rescue.

### 6.1 Poultry and swine lipidomic phenotypes

NDV infection remodels plasma phospholipid metabolism in chickens (Dai et al., 2024). This *in vivo* observation is valuable because it captures the integrated response of infected tissues, immune cells, circulation, and systemic metabolism. However, it does not by itself identify which lipids support viral replication. Follow-up studies should connect plasma changes to lipid composition in infected respiratory or lymphoid tissues, virions, and defined cell populations. Stable-isotope tracing and paired tissue–plasma sampling could distinguish local viral demand from systemic damage.

PRRSV-induced lipid-droplet accumulation and ASFV-induced cholesterol remodeling provide more direct links between cellular lipid reprogramming and replication (Yang et al., 2023; Chu et al., 2025). Even in these cases, broad metabolic inhibition is difficult to interpret. Reduced viral yield may reflect loss of membrane precursors, energy stress, altered innate signaling, or cytotoxicity. Rescue with specific lipids, genetic epistasis, and measurement of host-cell function are needed before a pathway can be considered a validated antiviral target.

### 6.2 Ferroptotic injury in JEV and influenza A virus infection

Ferroptosis is driven by iron-dependent peroxidation of susceptible phospholipids. In JEV infection, ferroptosis contributes to neuronal damage and neuroinflammation (Zhu et al., 2024). In influenza A virus-induced acute lung injury, epithelial ferroptosis is promoted by indoleamine 2, 3-dioxygenase 1 (Zheng et al., 2025). These studies shift oxidized phospholipids from biomarker status to a causal component of organ injury. They also create a therapeutic dilemma: oxidative lipid chemistry can influence viral replication and innate immunity, so tissue-protective ferroptosis inhibition must be evaluated together with viral load and antiviral signaling.

A related influenza A virus study showed that cytidine 5′-diphosphocholine corrected mitochondrial dysfunction in alveolar type II cells in infected mice (Doolittle et al., 2022). Such host-directed approaches may be valuable when tissue protection is clinically important, but they should be positioned as complements rather than assumed substitutes for direct antiviral control. The ideal intervention would protect the membrane function of vulnerable cells without creating a lipid environment that benefits viral replication.

## 7 Therapeutic strategies: from global lipid suppression to membrane-state editing

### 7.1 Target virus-specific lipid interfaces

Because phospholipids are essential, global depletion is rarely a realistic antiviral strategy. More selective opportunities include blocking a viral protein–lipid interaction, preventing viral recruitment of a lipid-transfer factor, disrupting a transient leaflet transition, or targeting a membrane state enriched only in infected cells or virions. The SVA 3C protease–phospholipid interface is an example of a potentially virus-selective target (Zhao et al., 2023). The NDV PS-externalization and acquisition mechanism suggests that preventing infection-induced PS externalization or receptor engagement could reduce progeny infectivity (Hou et al., 2025; Zhang et al., 2025). For ASFV, a recent study reported that rinderine suppresses ASFV replication in porcine alveolar macrophages by targeting membrane-associated profilin 1 and PE (Liu et al., 2025). Such findings require careful validation of selectivity, resistance barriers, pharmacokinetics, and effects on macrophage physiology.

Interventions directed at host lipid pathways should be designed around timing. An intervention may be effective only before attachment, during endosomal escape, during organelle expansion, or during budding. The study of lipid-raft dependence during IBV infection and the study of cholesterol flux during ASFV entry demonstrate that the same lipid pathway can have different importance across stages (Cuesta-Geijo et al., 2016; Guo et al., 2017). Time-of-addition experiments should therefore be treated as a minimum mechanistic requirement, not merely a pharmacological characterization.

### 7.2 Direct disruption of virion membranes for biosecurity applications

Virion envelopes offer another therapeutic and biosecurity target because they can be disrupted outside the host cell. A water-dispersible antimicrobial lipid mixture has been developed to inhibit ASFV and other enveloped viruses (Jackman et al., 2025). This approach is conceptually distinct from systemic lipid-metabolism inhibition: it acts directly on viral particles and may be suitable for environmental, feed, or water applications if efficacy, formulation stability, species safety, and organic-matter tolerance are established. Membrane-active agents should be evaluated against non-enveloped viruses and host membranes to define selectivity rather than being described as universally broad-spectrum. The stage-specific membrane features, supporting evidence, and potential translational implications discussed above are summarized in **Table 1**.

**Table 1:** Phospholipid logic across the life cycle of representative animal viruses.

Stage	Membrane feature	Representative animal-virus evidence	Evidence-supported role	Potential translational implication
Attachment/entry	Raft organization; cholesterol; endosomal BMP/LBPA	IBV, PRRSV, ASFV, influenza A virus (IAV; comparative mechanistic evidence)	Lipid domains and endosomal flux support attachment, internalization, endosomal progression, or fusion-pore formation.	Stage-specific entry inhibitors; virion-directed membrane disruption.
Replication	Lipid flux; phosphoinositides; lipid droplets; cholesterol synthesis	PRRSV, ASFV, SVA	Viral factories consume membrane material and can use lipid-stabilized protein conformations.	Block viral recruitment interfaces or viral protein–lipid binding.
Assembly/budding	PS/PE topology; envelope lipid phenotype	NDV, PRRSV	Producer-cell lipid asymmetry and envelope organization alter progeny infectivity.	Prevent PS-rich virion formation or receptor engagement.
Pathology	Peroxidizable phospholipids; ferroptosis	JEV, IAV, SVA	Oxidized phospholipids can drive neuronal or pulmonary injury and may also alter replication.	Combined antiviral and tissue-protective modulation.
Delivery/biosecurity	Engineered liposomes or membrane-active lipid mixtures	IBV, JEV, PEDV, IAV, ASFV	Lipids can serve as delivery vehicles or direct virion-disrupting agents.	Target-species vaccination, mucosal delivery, feed/water or environmental formulations.

### 7.3 Lipid-based delivery platforms: translational tools rather than evidence of lipid dependence

Liposomes, proliposomes, nanoliposomes, and cellular membrane nanovesicles can improve delivery of antivirals or vaccine antigens. Examples include proliposome-loaded ivermectin evaluated against IBV in embryonated chicken eggs (Alyami et al., 2025), CoPoP liposomes presenting JEV envelope antigen (Kim et al., 2026), cellular membrane nanovesicle-based vaccination against PEDV (Wang et al., 2026), and adjuvanted nanoliposomes for recombinant influenza A virus vaccines (Sia et al., 2026). These platforms are relevant to the Research Topic because they translate membrane engineering into animal-virus interventions. However, the delivery vehicle should not be conflated with a mechanistic claim that the virus depends on the same lipid pathway. A strong translational study should define cargo release, biodistribution, immune mechanism, stability, and safety in the target species.

For mucosal vaccination or local antiviral delivery, the relevant questions are formulation stability, epithelial access, cargo release, and target-species safety; these delivery considerations should remain conceptually separate from claims that a virus depends on a particular endogenous lipid pathway.

### 7.4 Protect tissue without increasing viral fitness

Ferroptosis inhibitors, membrane-repair modulators, and phospholipid precursors may reduce tissue injury, but their antiviral consequences are context dependent. Membrane repair involves PS and lipid-transfer proteins, including ORP-family pathways (Li et al., 2024). Enhancing repair could preserve epithelial or neuronal integrity, yet it might also maintain membranes needed for replication. Conversely, blocking repair could expose infected cells to immune clearance but worsen organ damage. Combined virological and pathological endpoints are therefore essential. The key experimental priorities for establishing causal roles of phospholipids and evaluating their translational relevance are summarized in **Table 2**.

**Table 2:** Experimental priorities for establishing causal roles of phospholipids and translational relevance.

Priority	Experimental question	Core approaches	Evidence for causality	Validation in target animal system
Spatial identity	Where is the relevant lipid change, and in which leaflet?	Organelle/virion isolation; imaging mass spectrometry; proximity-labeling lipidomics; topology-specific PS/PE probes	The localized lipid state precedes or tracks the defined viral phenotype; total abundance alone is insufficient.	Confirm in primary target cells and infected tissues; test species-specific lipid receptors where relevant.
Temporal causality	Which infection stage requires the membrane state?	Time-of-addition studies; acute degrons or inhibitors; live lipid sensors; lipid repletion	Acute perturbation blocks a defined stage, and compartment-appropriate rescue restores that stage.	Validate in species-relevant organoids or ex vivo tissues before challenge studies.
Reader identity	Which viral or host protein reads the lipid state?	Native-like bilayers; binding assays; structure-guided mutants; cryo-EM/NMR where feasible	A binding-defective mutant preserves folding and unrelated functions but loses the lipid-dependent phenotype.	Test relevant receptor/protein orthologs and evaluate viral fitness, transmission, and resistance in the natural host.
Lipid flux	Is lipid delivery, synthesis, or redistribution rate-limiting?	Stable-isotope pulse-chase; flux analysis; membrane-contact-site perturbation; live lipid sensors	Changing flux alters the relevant compartment and phenotype, with rescue by restoring the limiting route.	Confirm in natural target cells and tissues under physiologically relevant nutrient and differentiation conditions.
Translation	Can the intervention be used safely and practically?	PK/PD; toxicity; formulation stability; combination studies	Antiviral or tissue-protective effects are separated from cytotoxicity and generalized membrane failure.	Assess route, residue/withdrawal, cost, field stability, environmental impact, and vaccine compatibility.

## 8 Experimental priorities for establishing causality and translational relevance

### 8.1 Resolve the spatial membrane state

Future studies should combine mechanistic resolution with validation in species-relevant systems. The first priority is spatial resolution: investigators should determine the organelle, membrane domain, and leaflet in which a lipid change occurs. Total-cell lipidomics should be complemented by organelle or virion isolation, proximity-labeling lipidomics, imaging mass spectrometry, extracellular-leaflet probes, and direct virion lipid analysis. A bulk abundance change should not be assigned a mechanistic role until it is localized to the compartment that contains the relevant viral process.

### 8.2 Establish stage-specific causality

Causality must then be resolved in time. Acute and stage-specific perturbations are preferable to chronic knockout or prolonged inhibitor exposure, which can trigger compensatory lipid metabolism. Time-of-addition experiments, inducible degradation or inhibition, live lipid sensors, and defined lipid-repletion experiments should establish whether a membrane state is required for attachment, endosomal escape, replication-organelle expansion, assembly, or budding.

### 8.3 Validate in species-relevant systems

Mechanistic findings should progress from tractable cell lines to primary cells, organoids, ex vivo tissues, and, when justified, challenge models from the natural host. PRRSV studies should prioritize porcine alveolar macrophages; ASFV studies should validate findings in natural macrophage targets; and avian respiratory viruses should be tested in differentiated avian airway systems. Species-specific receptor orthologs, glycan patterns, and membrane composition should be measured rather than assumed from human or murine systems.

### 8.4 Identify the reader and demonstrate rescue

A proposed phospholipid mechanism should identify the reader and include a mechanistically specific rescue. If cholesterol perturbation blocks infection, restoring cholesterol to the relevant compartment should restore the affected stage without merely improving cell viability. If a viral protein is proposed to recognize a phospholipid, a binding-defective mutant should preserve folding and unrelated functions while losing the lipid-dependent phenotype. If lipid oxidation or ferroptosis is manipulated, viral replication, innate signaling, and tissue injury should be measured in parallel. These controls distinguish a phospholipid mechanism from generalized membrane stress.

### 8.5 Evaluate translational feasibility

Translational studies should determine whether the intervention retains a useful therapeutic index in the target species. Route and frequency of administration, pharmacokinetics and pharmacodynamics, formulation stability under field conditions, residue and withdrawal requirements for food animals, manufacturing cost, environmental impact, and compatibility with vaccination or biosecurity programs should be evaluated. A membrane target is not practically useful merely because it is mechanistically interesting.

## 9 Discussion

The phospholipid-code framework is intended as a testable organizational model rather than a claim that one universal lipid signature governs infection. Headgroup identity influences charge and protein recruitment; acyl-chain chemistry influences membrane mechanics and oxidative susceptibility; leaflet topology determines which extracellular or luminal sensors can access a lipid; and lipid flux supplies or remodels membranes. These variables are interpreted through functionally distinct readers—effectors, recruitment factors, and sensors. Consequently, the membrane requirements for entry, genome replication, assembly or budding, and tissue injury should be analyzed as separate, time-resolved states rather than collapsed into a single lipidomic profile.

Animal-virus examples demonstrate both commonality and specificity. NDV couples phospholipid transport to PS acquisition and enhanced progeny infectivity; PRRSV depends on raft organization in both cellular and viral membranes and redirects cellular lipid droplets; SVA uses a phospholipid as an allosteric regulator of its 3C protease; and JEV and influenza A virus link phospholipid peroxidation to tissue injury. Importantly, apparently similar cholesterol-related phenotypes are not equivalent: IBV shows a predominantly attachment-stage raft requirement in the Beaudette cell-culture system, whereas ASFV requires dynamic cholesterol flux during endosomal progression and later increases cholesterol supply or redistribution at replication sites. Such stage- and cell-type-specific requirements provide mechanistic hypotheses for differences in tissue tropism or host range, but direct causal tests in natural host tissues remain limited.

The framework also has important boundaries. Lipids act within a network that includes receptors, glycans, cytoskeletal motors, vesicular trafficking, signaling proteins, and organelle contacts. A lipid perturbation can therefore be causal, permissive, or merely correlative. Many available studies still rely on broad inhibitors, transformed cells, endpoint lipidomics, or total-cell measurements that do not resolve subcellular localization or leaflet exposure. Comparative human or plant virus studies can define transferable mechanisms, but they cannot substitute for validation in the natural animal host. Stronger evidence will require stage-resolved perturbation, rescue, structure-function analysis, and *in vivo* confirmation that separates antiviral activity from membrane toxicity.

The translational conclusion is therefore selective rather than expansive: not every infection-associated lipid change is a drug target. The most plausible opportunities are virus-specific protein-lipid interfaces, recruitment steps that are transiently required in infected cells, virion-membrane vulnerabilities accessible outside the host cell, and pathological lipid oxidation that can be modulated without increasing viral fitness. By explicitly defining the lipid species, compartment, leaflet, timing, reader class, cooperating host factors, and biological outcome, phospholipid virology can move from descriptive lipid lists toward falsifiable mechanisms and practical antiviral strategies for animal health.
